# A case of primary pleural synovial sarcoma with endobronchial recurrence

**DOI:** 10.1002/rcr2.547

**Published:** 2020-03-05

**Authors:** Jen Lye Wan, Yoke Fong Lam, Kit Weng Foong, Norsalwa Abdul Ghani, Kumaresh Lachmanan

**Affiliations:** ^1^ Department of Respiratory Hospital Raja Permaisuri Bainun Ipoh Malaysia; ^2^ Department of Anaesthesia Hospital Raja Permaisuri Bainun Ipoh Malaysia; ^3^ Department of Pathology Hospital Raja Permaisuri Bainun Ipoh Malaysia

**Keywords:** Endobronchial metastases, lung sarcoma, primary pleural synovial sarcoma

## Abstract

Primary pleural synovial sarcoma (PPSS) is an extremely rare malignancy without a known cause. The diagnosis is made after excluding metastasis from an extra‐thoracic sarcoma. We report a case of a 67‐year‐old gentleman who presented with an incidental finding of a left lung mass on a routine chest X‐ray. A computed tomography (CT) of the thorax and whole‐body positron emission tomography (PET)‐CT was done confirming a left lung mass with no other extra‐thoracic involvement. A lobectomy was performed with a diagnostic and therapeutic intent. The histopathological examination and immunohistochemistry study revealed a pleural‐based tumour with features suggestive of synovial sarcoma. Subsequently, he underwent post‐operative radiotherapy. However, three months later, he developed an endobronchial recurrence, complicated by post‐obstructive pneumonia resulting in his demise. This case highlights a rare form of malignancy with a rare site of recurrence.

## Graphical abstract

Primary pleural synovial sarcoma (PPSS) is an extremely rare malignancy without a known cause. We report a case of a PPSS that was operated and recurred as an endobronchial metastasis.

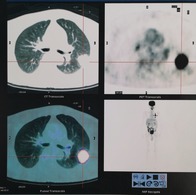

## Introduction

In general, synovial sarcoma, a malignant soft tissue tumour, comprises up to 10% of soft tissue sarcomas. It is a mesenchymal spindle cell tumour with variable epithelial differentiation. The lung is the usual metastasis site for soft tissue synovial sarcoma. However, this tumour can arise as a primary lesion in other parts of the body such as the lung, pleura, and chest wall. Primary synovial sarcomas of the pleura are extremely rare. Most patients present between the age of 15 and 35 years, and 90% of cases are reported before 50 years of age 1. It affects both genders equally 2. We report a case of a primary pleural synovial sarcoma (PPSS) that was operated and recurred as an endobronchial metastasis.

## Case Report

A 67‐year‐old Chinese man with underlying comorbidity of diabetes mellitus was referred to the pulmonology clinic after a chest X‐ray (CXR) done in January 2019 (workup prior to elective hernioplasty) showed an incidental finding of a left pulmonary mass. This gentleman had no respiratory or constitutional symptoms. Physical examination was unremarkable and his tumour markers were all within normal limits.

**Figure 1:**
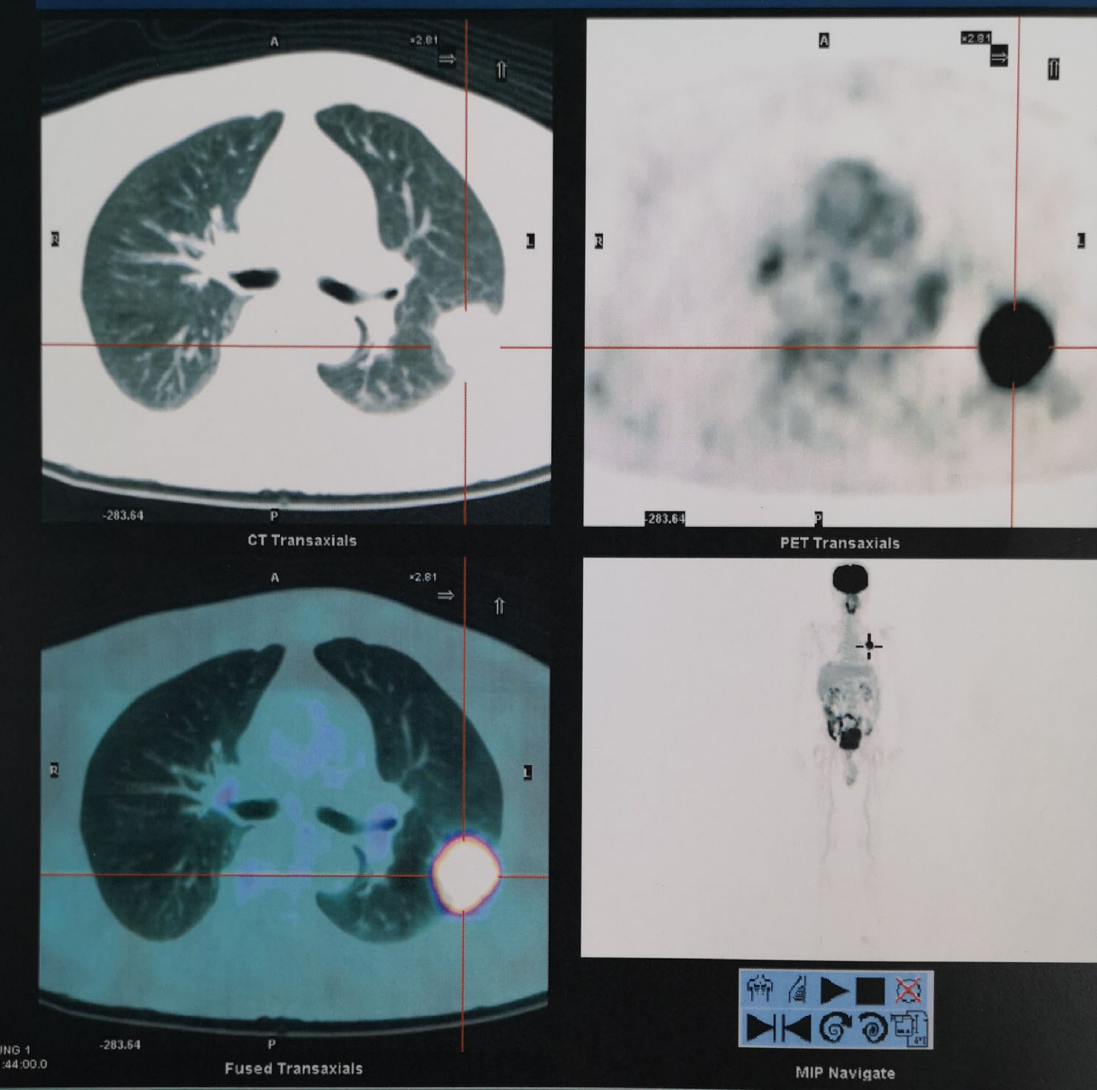
Positron emission tomography of the thorax. A lobulated FDG‐avid mass was seen at the left upper lobe (SUVmax: 20.2) with a metabolic size of 3.7 × 3.0 × 3.8 cm. No other FDG‐avid nodule/mass was seen at the remaining left lung parenchyma or at the right lung parenchyma. No FDG‐avid nodal metastasis or evidence of distant metastasis was seen.

A contrast computed tomography (CT) of the thorax showed a lobulated mass of 3.4 × 2.7 × 2.6 cm in the posterior segment of the left upper lobe. Positron emission tomography (PET)‐CT showed a lobulated FDG‐avid mass in the left upper lobe with metabolic size of 3.7 × 3.0 × 3.8 cm without any evidence of distant lesions (Fig. 1). A CT‐guided biopsy was done suggesting a high‐grade myogenic sarcoma.

**Figure 2:**
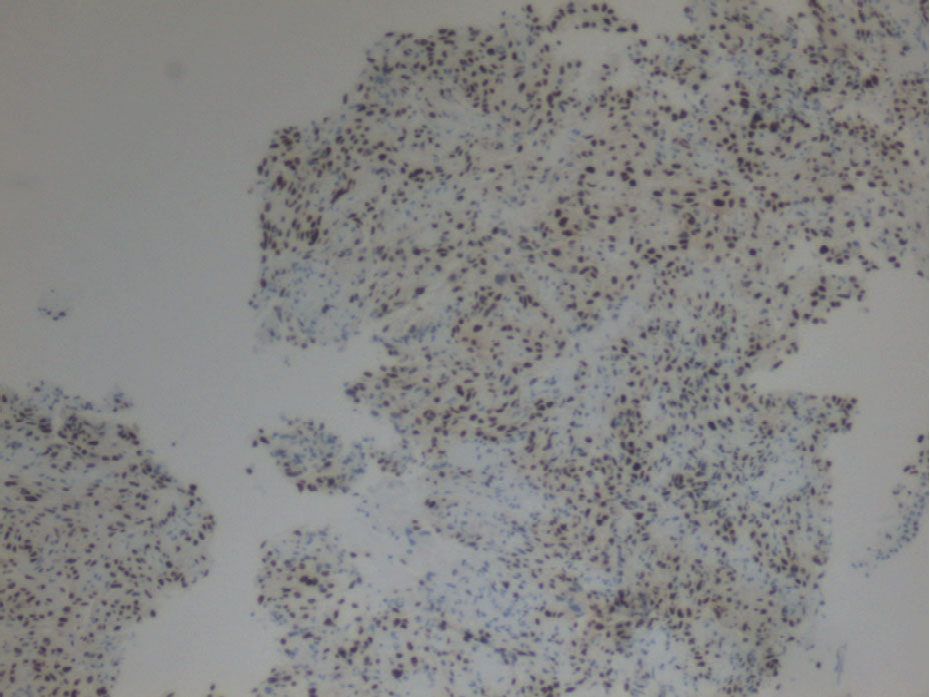
Histology: The nuclear cells were of strong nuclear positivity towards transducer like enhancer of split 1 (TLE1), epithelial membrane antigen staining (EMA), and cytokeratin MNF 116 (MNF116), and <10% of the spindle cell component was positive towards S100 protein which excluded malignant peripheral nerve sheath tumour. It was negative to cluster of differentiation (CD)34, human melanoma black (HMB)45 (melanocytic marker), and CD117 [marker for gastro intestinal stromal tumour (GIST)].

He underwent left upper lobectomy that suggested a poorly differentiated PPSS on histological and immunohistochemistry (IHC) studies (Fig. 2).

Post‐operatively, a repeat PET‐CT in March 2019 revealed only fluorodeoxyglucose (FDG) avidity at the right hilar region [standardized uptake values (SUVmax): 3.4], but no obvious nodule was seen on low‐dose non‐contrast CT. He was then treated with radiotherapy to the previous operative site after an oncology consult. No subsequent surveillance imaging was performed.

In September 2019, he presented with an acute history of fever, cough, and tachypnoea for three days. His blood investigations showed a raised white blood cell (WBC) count of 30.7 × 10^9^, Hb 11.4 g/dL, an elevated C‐reactive protein 318 g/dL, and erythrocyte sedimentation rate (ESR) of 73. The CXR on admission showed a left mid‐zone opacity with air bronchogram. Physical examination only revealed coarse crepitations over the left lower zones.

**Figure 3:**
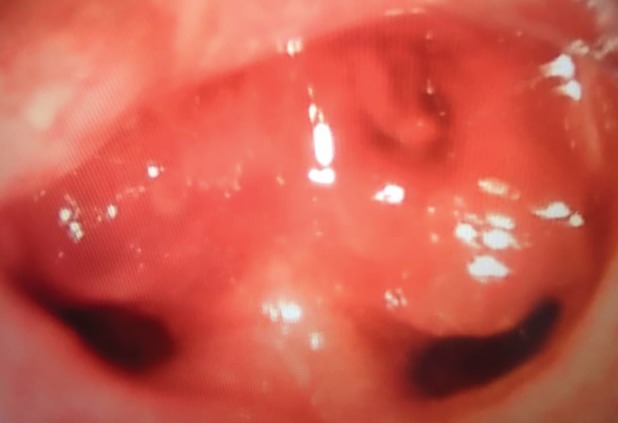
An endoscopic view of main carina with endobronchial metastasis.

He required endotracheal intubation and mechanical ventilation and was treated as pneumonia with intravenous antibiotics. A bronchoscopy found a fleshy growth at the main carina, obstructing both the right and left main bronchi (Fig. 3). Multiple biopsies were done, for which the histopathology was reported as a metastatic synovial sarcoma. An urgent CT of the thorax and abdomen showed irregular peripherally enhancing lesions in the mediastinum with local extensions into the carina compressing bilateral main bronchus and ill‐defined liver nodules were suggestive of metastases. An urgent referral was made for tumour debulking and stenting of the airway, but the patient's family members did not approve further intervention. After a week in intensive care unit (ICU), the patient succumbed to sepsis and disease progression.

## Discussion

Synovial sarcoma, a rare mesenchymal tumour, accounts for 10% of all soft tissue sarcomas. Among pulmonary malignancies, PPSS constitutes 0.1–0.5%.

The clinical symptoms of PPSS vary widely, mainly based on the histology and region of the tumour occurrence, as well as size and degree of tumour differentiation 3. Initially, the patient may be asymptomatic but as the disease progresses, patients may present with non‐specific pulmonary symptoms such as chest pain, cough, or dyspnoea while our patient, on disease recurrence, presented with sepsis caused by post‐obstructive pneumonia.

Four histological subtypes have been described: biphasic, monophasic (spindle), monophasic epithelial, and poorly differentiated. The two most common are the monophasic (spindle) and biphasic subtypes 3. However, our patient had poorly differentiated synovial sarcoma. The initial histology showed spindle cell hypercellular proliferation in fascicular pattern, in which the spindled tumour cells have ample eosinophilic cytoplasm that resembled smooth muscle cells with marked nuclear pleomorphism. Ancillary IHC staining technique is crucial in making the final diagnosis and to exclude other possible differentials. It has been recently suggested that vimentin, cytokeratin, and epithelial membrane antigen staining (EMA) in combination with CD34 negativity are the most useful and sensitive protein biomarkers for the diagnosis of monophasic fibrous synovial sarcoma and poorly differentiated synovial sarcoma 3.

There is currently no standardized therapy for patients with primary pulmonary synovial sarcoma, and complete surgical resection remains the main strategy in these patients 3. These tumours are highly aggressive, and the overall prognosis is poor with an overall five‐year survival rate of 50%. In advanced or unresectable tumours, doxorubicin‐ and ifosfamide‐based chemotherapy can be used, with an overall response rate of around 24% 4. A new drug pazopanib (tyrosine kinase inhibitor) seems to provide another option with an improved median progression‐free survival in some trials 5. Radiotherapy has no apparent effect on the control of local disease or overall survival.

For our patient, we initially planned for tumour debulking and stenting of the airway as a management of the post‐obstructive pneumonia. Should he have recovered from his pneumonia, our further management would be proceeding with doxorubicin‐ and ifosfamide‐based chemotherapy depending on his clinical performance status. However, the patient's family members opted for conservative management and the patient subsequently succumbed to disease progression.

### Disclosure Statement

Appropriate written informed consent was obtained for publication of this case report and accompanying images.
